# Clinical outcomes of diverticular disease in young adults: results from a tertiary referral center

**DOI:** 10.3389/fmed.2024.1363548

**Published:** 2024-04-04

**Authors:** Giovanni Santacroce, Marco Vincenzo Lenti, Giulia Maria Abruzzese, Giacomo Alunno, Francesco Di Terlizzi, Carmine Frenna, Antonella Gentile, Mario Andrea Latorre, Clarissa Petrucci, Damiano Ruggeri, Simone Soriano, Nicola Aronico, Annalisa De Silvestri, Gino Roberto Corazza, Marietta Iacucci, Antonio Di Sabatino

**Affiliations:** ^1^Department of Internal Medicine and Medical Therapeutics, University of Pavia, Pavia, Italy; ^2^First Department of Internal Medicine, San Matteo Hospital Foundation, Pavia, Italy; ^3^Scientific Direction, Clinical Epidemiology and Biometric Unit, San Matteo Hospital Foundation, Pavia, Italy; ^4^APC Microbiome Ireland, College of Medicine and Health, University College of Cork, Cork, Ireland

**Keywords:** diverticula, hospitalization, outcome, prevention, recurrence, risk factor

## Abstract

**Introduction:**

Diverticular disease (DD), commonly associated with the elderly, is becoming more prevalent among younger individuals. This retrospective study aimed to evaluate the differences in the natural history and outcomes between young and old patients with DD.

**Methods:**

Adult patients with DD diagnosed between 2010 and 2022 at an Italian tertiary referral center were enrolled, and their demographic and clinical data were retrieved. The patients were categorized as young or old based on the 25th percentile of the population's age at diagnosis. Univariate and multivariate analyses were performed to assess the association between the collected variables and the age of disease presentation. Additionally, survival analyses were conducted to evaluate the association between the age of diagnosis and clinical outcomes at follow-up, including disease recurrence, hospital access, surgery, and death.

**Results:**

A total of 220 DD patients (with a median age of 66 years, IQR 55–74, and a female-to-male ratio of 1.4:1) were included in the study, comprising 54 patients receiving a diagnosis before the age of 49 years (young DD patients) and 166 patients diagnosed after the age of 49 years (old DD patients). Male sex (57 vs. 36%, *p* < 0.01), smoking (38 vs. 14%, *p* < 0.01), and alcohol consumption (54 vs. 38%) were highly prevalent in young patients. The complications at the time of diagnosis, particularly abscesses and free perforations, occurred more frequently in younger patients (*p* = 0.04). Moreover, young DD patients experienced a higher rate of hospitalization and surgical intervention (*p* = 0.01 and *p* = 0.04, respectively) over a median follow-up period of 5 years.

**Conclusion:**

Preventive strategies and prompt diagnosis are crucial in young patients with DD for achieving better disease outcomes and preventing complications.

## 1 Introduction

Diverticular disease (DD), considered as one of the most common gastrointestinal disorders among inpatients and outpatients (1), entails a spectrum of manifestations which depend on colonic diverticulosis. According to the European Society of Coloproctology, this spectrum encompasses symptomatic uncomplicated diverticular disease (SUDD) and symptomatic complicated disease (2). The latter includes acute diverticulitis, which may be uncomplicated or complicated if the inflammatory process extends beyond the colonic wall, leading to abscess and perforation. If left unresolved, acute diverticulitis can advance to chronic diverticulitis, which may be uncomplicated or complicated by stenosis and fistulation. Additionally, the spectrum also includes diverticular bleeding, a consequence of ruptured diverticular vessels. More recently, the spectrum has incorporated a rare entity named segmental colitis associated with diverticulosis (SCAD), which shares multiple clinical and histologic features with inflammatory bowel disease (3).

The global prevalence of DD is increasing, particularly in Western countries. The reported prevalence stands at 12–22 cases per million individuals in the United States and 8–12 cases per million individuals in Western Europe (4). Although historically more prevalent among elderly patients (>65 years), a substantial surge in its prevalence has been observed in young adults (< 40 years) (5). Indeed, diverticulosis has shown a significant rise in the incidence among individuals aged 18–44 years, escalating from 0.15 to 0.251 per 1,000 persons within just 7 years (5). Similarly, a surge in the incidence of acute diverticulitis has been reported among individuals aged 40–49 years, with a 132% increase from 1980 to 2007 (6). Notably, younger patients with diverticulosis exhibit a substantially higher annual incidence of diverticulitis compared to older patients (7).

Several risk factors contribute to diverticular disease, comprising both modifiable and non-modifiable factors. The non-modifiable factors encompass age, sex, and genetics. The modifiable factors include dietary habits, particularly fiber intake, along with physical activity, smoking, alcohol consumption, and medication use, such as non-steroidal anti-inflammatory drugs and opiates (1). However, the specific risk factors associated with the diagnosis of the disease at a younger age remain unclear. Furthermore, whether disease onset at a younger age entails distinct clinical manifestations and worse long-term outcomes requires further elucidation. In other gastrointestinal inflammatory disorders, such as Crohn's disease and ulcerative colitis, significant differences in the natural history of the disease and its outcomes have been observed between early and late-onset cases (8, 9). Hence, characterizing the profile of young patients with DD could be equally crucial, as it holds the potential in enabling the implementation of proactive prevention strategies, timely diagnosis, and appropriate therapeutic approaches.

Although the optimal therapeutic management of diverticular disease remains a topic of debate, several therapeutic approaches have been explored (4). For SUDD, strategies such as the use of poorly absorbed antibiotics, anti-inflammatory drugs like mesalamine, and probiotics have been proposed. Additionally, emerging non-conventional approaches for SUDD include the use of the medicinal fungus *Hericium erinaceus* and nutraceutical formulations (10, 11). In the case of diverticulitis, antimicrobial therapy is often recommended, particularly for high-risk disease, while surgical intervention may be necessary for managing complications.

Building upon these premises, this retrospective study primarily aims to analyse differences in demographic, clinical, and prognostic features between young and old patients with DD.

## 2 Materials and methods

### 2.1 Study population and design

This single-center retrospective study was conducted at a tertiary referral center in Northern Italy (San Matteo Hospital Foundation). Adult patients (>18 years of age) diagnosed with DD (including SUDD, acute diverticulitis, chronic diverticulitis, or SCAD) between 2010 and 2022 were selected to be included in the study. To ensure accurate inclusion, diagnoses were retrospectively reassessed based on the recent international guidelines (2), and patients with unconfirmed DD diagnosis were excluded.

For SUDD, patients with persistent abdominal pain, particularly in the lower left abdomen, and any imaging evidence of colonic diverticula were included. Diverticulitis was diagnosed in patients with abdominal symptoms accompanied by evidence of peridiverticular inflammation on cross-sectional imaging and laboratory tests. Diverticulitis was further categorized as acute or chronic based on the complete resolution of the acute event. Additionally, it was classified as uncomplicated or complicated depending on the presence of complications, such as abscess, fistula, obstruction, and free perforation. The diagnosis of SCAD was established only in patients with localized inflammation in the colon segments affected by diverticulosis, sparing the diverticular orifice and the rectum, and confirmed by histology.

In order to retrieve the largest amount of data and prevent any potential diagnostic and data collection biases, we exclusively included in the analysis those DD patients who had visited the gastroenterological outpatient department at least once recently (since 2018, which is the 1st year when digital reports became available in our center). Patients without a recent clinical follow-up were excluded. Additionally, telephonic follow-up interviews were conducted in May 2023, and patients who did not take part in this interview were excluded from the analysis.

All patient data were extracted from medical records, and missing data were retrieved through the telephonic follow-up.

The primary endpoint of the study was to identify risk factors and different clinical presentations associated with the presentation of DD at a younger age. Patients were classified into the young and old groups based on the 25th percentile of age at disease presentation. As a secondary aim, we assessed the clinical outcomes of young DD patients compared to those of elderly patients.

### 2.2 Sociodemographic and clinical data

For each patient, comprehensive sociodemographic and clinical data were collected, including age (years), sex, family history of DD, smoking habits (i.e., active smokers, irrespective of the number of cigarettes/day, and individuals who quit smoking for < 5 years), fiber intake (< 10 estimated total grams of fiber per day was considered as low dietary fiber intake), alcohol consumption (more than 3.5 alcohol units/day for men and 1.75 alcohol units/day for women), bowel movements, comorbidities (including any clinically significant comorbidities—i.e., neoplastic, gastrointestinal, cardiovascular, neurological, and rheumatological disorders that require specific treatment and impact patient outcomes—and all cardiovascular and gastrointestinal neoplastic comorbidities), previous use of non-steroidal anti-inflammatory drugs, steroids, and opiates, history of abdominal surgery, exercise habit (at least 2 h of moderate physical activity/week, assessed through the International Physical Activity Questionnaire), and body mass index (Kg/m^2^). All these variables were selected in accordance with the available literature on this topic. Disease-related details at diagnosis were evaluated, i.e., the type of DD (SUDD, acute diverticulitis, chronic diverticulitis, and SCADD), diverticula localization (sigmoid colon and multiple localization), complications (including abscess, fistula, obstruction, and free perforation), and the need for hospitalization or surgery. DD features were assessed through imaging modalities, including computed tomography scan, intestinal ultrasound, and endoscopy.

### 2.3 Follow-up data

Follow-up data, including the clinical outcomes such as disease recurrence, hospitalization, need for surgery, and death associated with diverticular disease, were assessed using the recent medical records and through telephonic interviews conducted with the patients.

### 2.4 Statistical analysis

The statistical analysis was conducted using Stata 17 (StataCorp, College Station, TX, USA). We used medians and interquartile ranges (IQRs) to describe continuous data, while categorical data were presented as counts and percentages. The normality of the distributions was assessed using the Shapiro–Wilk test. The missing data were excluded from statistical calculations. The chi-squared test and Mann–Whitney test were employed to assess the association between the 25th percentile of age at diagnosis (young vs. old DD patients) and the relevant variables. A two-sided *p*-value < 0.05 was considered statistically significant. The multivariate analysis was performed using logistic regression models, and odds ratios were expressed as exponential of the β-value. The McFadden pseudo *R*^2^ was used to establish the goodness of fit of the model. The Kaplan-Meir method and the log-rank test were used for survival analysis.

The study was approved by the local ethics committee (2016, Protocol number 004820), and patients provided written informed consent prior to study participation.

## 3 Results

### 3.1 Patients enrolled and stratification according to young vs. old DD patients

**Figure 1:**
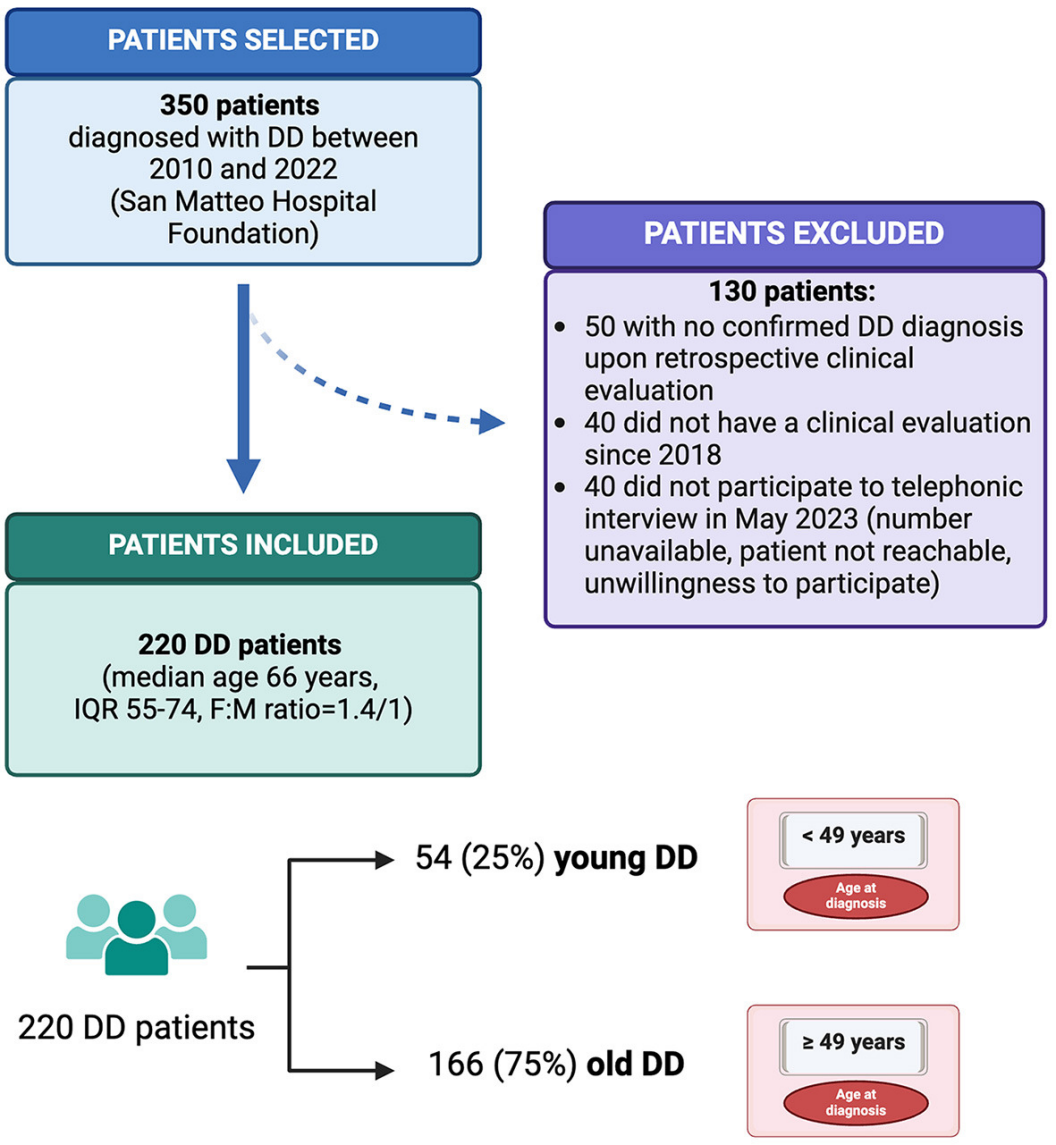
Flowchart of the patient enrolment and inclusion process. This figure illustrates the process of retrospective patient enrolment and inclusion in the study. The two study groups, young and old diverticular disease patients, are represented. DD, diverticular disease; F, female; IQR, interquartile range; M, male. Created with “Biorender.com”.

A total of 350 patients with a previous diagnosis of DD were retrospectively enrolled in the study. According to the inclusion criteria, 220 patients (with a median age of 66 years, IQR 55–74, and a female-and-male ratio of 1.4:1) were eventually considered in the analysis. Figure 1 shows the flowchart of the enrolment and inclusion of patients in the study analysis. In Table 1, the sociodemographic and clinical data of study population at diagnosis are reported. Overall, 124 patients (56%) had SUDD, 74 (34%) had acute diverticulitis, 16 (7%) had chronic diverticulitis, and 6 (3%) had SCAD. The main localization of diverticula was the sigmoid colon, with 37% of the patients showing multiple localization. Additionally, 40 patients (23%) were hospitalized for DD, with 19 (9%) of them having complications (namely eight abscesses, two fistulas, one bowel obstruction, and nine free perforation) and 8 (5%) of them requiring surgery at diagnosis.

**Table 1:** Sociodemographic and clinical features of the study population at the time of diagnosis with the univariate analysis for factors affecting diverticular disease diagnosis in young vs. old patients.

	**Overall DD patients**	**Young DD patients (< 49 y)**	**Old DD patients (≥49 y)**	***p***
Patients *n* (%)	220 (100)	54 (25)	166 (75)	-
Age (median) [range], years	66 [55–74]	46 [41–55]	70 [63–78]	-
F/M (ratio)	129/91 (1.4/1)	23/31 (1/1.34)	106/60 (1.8/1)	**< 0.01**
Family history of diverticular disease (%)	32 (20)	11 (25)	21 (18)	0.35
Smoking habit (%)	34 (21)	18 (38)	16 (14)	**< 0.01**
Low dietary fiber intake (%)	17 (12)	3 (7)	14 (13)	0.33
Alcohol consumption (%)	60 (42)	21 (54)	39 (38)	**0.03**
Bowel movements (%)				
Constipation	54 (29)	13 (26)	41 (30)	0.95
Diarrhea	47 (25)	13 (26)	34 (25)	
Mixed	34 (18)	10 (20)	24 (18)	
Comorbidities (%)				
Any clinically significant	135 (61)	21 (39)	114 (69)	**< 0.01**
CV	97 (44)	14 (26)	83 (50)	**< 0.01**
GI	166 (76)	38 (70)	128 (78)	0.28
GI neoplasms	26 (13)	1 (2)	25 (16)	**< 0.01**
Previous NSAIDs/steroid use (%)	45 (27)	7 (17)	38 (30)	0.09
Previous opiates use (%)	5 (3)	0 (0)	5 (4)	0.41
Previous abdominal surgery (%)	82 (45)	16 (34)	66 (48)	0.09
Exercise (%)	66 (52)	20 (55)	46 (50)	0.81
BMI (median) [IQR], Kg/m^2^	24 [22–27]	24 [22–28]	25 [22–27]	0.65
Type of DD (%)				
SUDD	124 (56)	26 (48)	98 (59)	0.39
Acute diverticulitis	74 (34)	52 (31)	22 (41)	
Chronic diverticulitis	16 (7)	5 (9)	11 (7)	
SCAD	6 (3)	1 (2)	5 (3)	
Diverticula localization at diagnosis (%)				
Sigmoid colon	101 (55)	22 (45)	79 (58)	0.35
Multiple localization	68 (37)	20 (41)	48 (35)	
Hospitalization at diagnosis (%)	40 (23)	14 (30)	26 (20)	0.17
Complications at diagnosis (%)	19 (9)	9 (18)	10 (6)	**0.04**
Abscess	8 (4)	3 (6)	5 (3)	
Fistula	2 (1)	0 (0)	2 (1)	
Obstruction	1 (1)	0 (0)	1 (1)	
Free perforation	9 (5)	6 (12)	3 (2)	
Need for surgery at diagnosis (%)	8 (5)	3 (7)	5 (4)	0.48

BMI, body mass index; CV, cardiovascular; DD, diverticular disease; F, female; GI, gastrointestinal; IQR, interquartile range; M, male; NSAID, non-steroidal anti-inflammatory drug; SCAD, segmental colitis associated with diverticulosis; SUDD, symptomatic uncomplicated diverticular disease. Missing data were excluded from percentage calculation. In bold, variables reaching statistical significance (*p* < 0.05).

When considering the 25th percentile of age at disease presentation, two groups were defined, namely young DD patients (54; 25%), who were diagnosed before the age of 49 years, and old DD patients (166, 75%), who were diagnosed after the age of 49 years. The patient stratification is shown in Figure 1.

### 3.2 Univariate analysis for factors affecting DD diagnosis in young vs. old individuals

In Table 1, the results of the univariate analysis for factors affecting DD diagnosis in young vs. old patients are reported. The male sex resulted a significant risk factor for younger age at DD presentation (*p* < 0.01). Similarly, smoking and alcohol consumption were highly prevalent in younger patients (*p* < 0.01 and *p* = 0.03, respectively) compared to the older patients. Conversely, clinically significant comorbidities, cardiovascular comorbidities, and a history of gastrointestinal tract neoplasia were significantly (*p* < 0.01) more frequent in elderly patients, being collinear with age. No significant difference was found regarding the specific type or localization of DD. The complications at diagnosis, particularly abscess (6 vs. 3%) and free perforation (12 vs. 2%), occurred more frequently in young DD patients compared to old DD patients (*p* = 0.04). Moreover, younger DD patients showed a higher, despite non-significant, rate of hospitalization (30 vs. 20%) and need for surgery (7 vs. 4%) at diagnosis.

### 3.3 Multivariable analysis for factors affecting DD diagnosis in young vs. old patients

Table 2 shows the results of multivariable analyses for factors affecting the age of disease presentation. The male sex proved to be a risk factor for diagnosis at a younger age (OR 2.55, 95% CI: 1.013–6.434, *p* = 0.04). Moreover, the smoking habit was identified as another risk factor associated with the diagnosis of DD in young patients (OR 3.02, 95% CI: 1.059–8.629, *p* = 0.03). No significant associations were observed for the other variables considered.

**Table 2:** The multivariate analysis for factors affecting diverticular disease diagnosis in young vs. old patients.

**N. Obs = 130 (Young-DD = 40—Old-DD = 90)**	**β**	**Odds ratio**	**95% CI**	***p***
Sex (M = 53—F = 77)	0.94	2.55	1.013 – 6.434	**0.04**
Smoking habit (Yes = 25—No = 105)	1.11	3.02	1.059 – 8.629	**0.03**
Alcohol consumption (Yes = 55—No = 65)	0.04	1.04	0.419 – 2.562	0.19
Previous NSAIDs/steroids use (Yes = 34—No = 96)	−0.97	0.38	0.119 – 1.209	0.10
Previous abdominal surgery (Yes = 57—No = 73)	−0.28	0.75	0.297 – 1.933	0.56

Pseudo R^2^ = 0.25. CI, confidence interval; DD, diverticular disease; F, female; M, male; NSAID, non-steroidal anti-inflammatory drug; Obs, observation. In bold, variables reaching statistical significance (*p* < 0.05).

### 3.4 Follow-up and young vs. old DD patients

**Figure 2:**
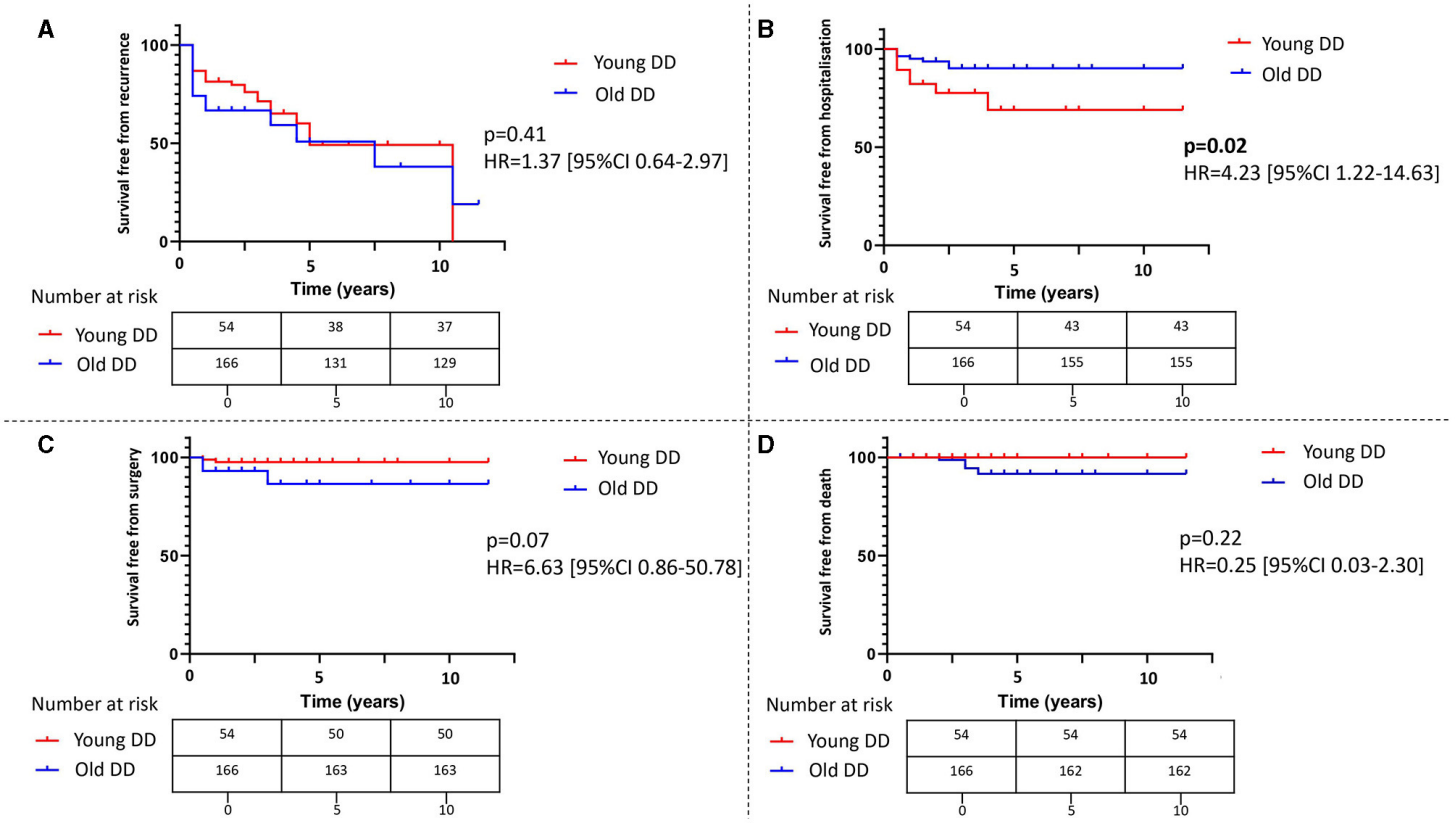
The survival analysis for young vs. old diverticular disease patients. The figure shows the Kaplan-Meier curves evaluating differences in survival free from recurrence **(A)**, hospitalization **(B)**, surgery **(C)**, and death **(D)** in patients with diverticular disease according to the age of diagnosis. The differences were compared using the log-rank test, and a two-sided *p*-value < 0.05 was considered statistically significant. Hazard ratios with 95% confidence intervals and tables with number at risk are provided. DD, diverticular disease; HR, hazard ratio; CI, confidence interval.

At follow-up (median of 5 years, IQR 4-9), 25% of the patients experienced disease recurrence, 11% required hospitalization, and a minority underwent surgery or died because of DD (see Table 3). When stratifying by young vs. old DD patients, a significantly higher frequency of hospital access (*p* = 0.01) and need for surgery (*p* = 0.04) was seen in younger patients. Additionally, a higher, despite not statistically significant, rate of disease recurrence was seen in younger patients (33 vs. 23%). In the survival analysis, young DD patients exhibited a reduced survival free from hospitalization (HR 4.23, 95% CI: 1.22–14.63; *p* = 0.02), while no statistically significant differences were observed for other reported outcomes. The Kaplan-Meier curves depicting these findings are shown in Figure 2.

**Table 3:** Follow-up data of young and old diverticular disease patients.

	**Overall patients**	**Young DD patients (< 49 y)**	**Old DD patients (≥49 y)**	***p***
Patients *n* (%)	220 (100)	54 (25)	166 (75)	-
F.U median (years) [IQR]	5 [4–9]	5 [3–9]	5 [4–8]	-
Disease recurrence (%)	56 (25)	18 (33)	38 (23)	0.12
Hospital access (%)	24 (11)	11 (20)	13 (8)	**0.01**
Need for surgery (%)	7 (3)	4 (7)	3 (2)	**0.04**
Death (%)	4 (2)	0 (0)	4 (2)	0.25

DD, diverticular disease; F.U, follow-up; IQR, interquartile range. In bold, variables reaching statistical significance (*p* < 0.05).

## 4 Discussion

Our retrospective single-center study compared young and elderly patients affected by DD. We identified two patient groups: young DD patients under the age of 49 years (25th percentile of age at diagnosis within our population) and older DD patients over the age of 49 years. We observed a higher prevalence of male sex, smoking habit, and alcohol consumption among young patients. Furthermore, young patients showed more complications at diagnosis, especially abscesses and free perforations, and had worse outcomes during the follow-up, including a higher rate of hospitalization and surgical intervention.

The observed prevalence in male sex of the young DD group aligns with the recent epidemiological evidence suggesting a higher incidence in male individuals up to the age of 50 years, while the condition tends to be more prevalent in female individuals among older patients (12). In addition to sex, two additional risk factors were significantly more prevalent in young DD patients, namely smoking habits and alcohol consumption, although the latter was not confirmed in the multivariate analysis. Smoking is a well-established risk factor for DD, with an odds ratio of 1.23–1.89 for acute diverticulitis, whereas the role of alcohol as a risk factor remains under discussion (1). The results of our multivariate analysis warrant caution due to the limitations posed by the sample size and the wide confidence intervals of odds ratios. Nonetheless, identifying these two modifiable risk factors as prevalent in young DD patients highlights the importance of proactive preventive strategies targeting the lifestyle of younger individuals, with the aim of reducing the incidence and prevalence of this disabling disease (13). Additionally, these factors could serve to help the diagnostic reasoning of physicians. In young patients presenting clinical features consistent with DD, especially for more subtle and insidious forms such as SUDD, these factors can aid in achieving a prompt and accurate diagnosis. This can be facilitated through the appropriate use of available clinical diagnostic tools such as the fecal calprotectin test and intestinal ultrasound scans (14, 15). Timely diagnosis holds paramount importance in reducing the diagnostic delay associated with this condition, which was recently reported to have a median of 7 months in SUDD (16). A reduced diagnostic delay can lead to a decreased onset of complications both at diagnosis and follow-up (16).

The existing data concerning the course of DD in young patients are conflicting (17). While historically, younger age was considered a risk factor for a more aggressive DD with a severe clinical course and increased complication rate (18), recent evidence has shown contrasting findings (19). In recent systematic reviews and meta-analyses, young DD patients did not show a more aggressive or complicated disease course, although a higher recurrence rate was observed (20–22). Van Dijk et al. argued about the association between age and the recurrence of diverticulitis, reporting a higher RR of 1.47 (95% confidence interval 1.20–1.80) for young patients based on raw recurrent diverticulitis rates. However, they found no association in studies using survival analyses and considering the duration of follow-up within age groups (23).

Given these inconsistencies, our study aimed to examine the disparities between young and old DD patients, focusing on their distinct clinical presentations and outcomes over a median 5-year follow-up. Unlike previous studies focusing solely on diverticulitis, our study is the first to encompass the entire spectrum of DD, including SUDD, acute diverticulitis, chronic diverticulitis, and SCAD.

In our cohort, we observed no difference between young and elderly DD patients regarding the different forms of DD or diverticula localization. However, young patients exhibited a higher incidence of hospitalization (30 vs. 20%) and surgery (7 vs. 4%) at diagnosis, despite not reaching statistical significance. Additionally, there was a significant increase in the number of complications (18 vs. 6%), particularly abscesses and free perforations. These findings align with a previous prospective study showing a higher complication rate in young DD patients (18). Our study also revealed differences in the outcomes of young DD patients, demonstrating through survival analysis a significantly lower rate of hospitalization-free survival for this group (*p* = 0.02). Additionally, younger patients experienced a higher disease recurrence rate (33 vs. 23%) and a significant increase in hospital admissions (20 vs. 8%, *p* = 0.01) and need for surgery (7 vs. 2%, *p* = 0.04) compared to elderly patients during the follow-up period. All these findings seem to point at a more aggressive disease phenotype at diagnosis in younger DD patients, along with a heightened risk of long-term complications.

Currently, guidelines do not advocate for distinct therapeutic approaches for young patients, recommending no deviation in medical or surgical management (1, 2, 6). However, our findings suggest a distinct natural history among young patients with DD, characterized by a heightened risk of complications both at baseline and follow-up. Therefore, a more proactive therapeutic approach should be considered for young DD patients to avert complications and reduce the disease burden on patients and society. In the case of diverticulitis in young patients, the evaluation for potential complications via cross-sectional imaging and diagnostic laparoscopy should be considered to ascertain their occurrence and the need for intravenous antibiotics and/or surgical intervention. In the absence of complications, guidelines suggest a personalized approach, potentially involving antibiotics and anti-inflammatory drugs (1). Our data highlight that young age should be taken into account as a risk factor for a more severe disease course when making therapeutic decisions (1). Furthermore, in patients with a prior diverticulitis episode or with SUDD, the active prevention of diverticulitis and the maintenance of disease remission should be considered the primary clinical goals in young individuals. While the optimal management is still matter of debate, a long-term strategy involving antibiotics (particularly poorly absorbed antibiotics such as rifaximin) or anti-inflammatory drugs (such as mesalamine) and probiotics may be recommended (4). Additionally, from a personalized approach standpoint, elective resection following an acute episode of diverticulitis in young patients could be considered. However, addressing this matter requires further investigation as recent meta-analyses have failed to define the timing of elective surgery in young patients with DD (24).

Our study has some limitations that need to be mentioned. First, it is a retrospective study and, therefore, suffers from the bias associated with data collection, as not all data were consistently available in clinical records. We attempted to address this limitation by selecting patients with recent follow-up and retrieving missing data through telephone calls. However, there were instances where certain data, such as the Diverticular Inflammation and Complication Assessment (DICA) classification or the Combined Overview on Diverticular Assessment (CODA) score (25, 26), could not be retrieved. These objective parameters would have been valuable for comparing the two groups, particularly in terms of outcomes. Furthermore, this is a single-center study involving a selected population evaluated in a tertiary referral hospital, which may have introduced a selection bias. Therefore, larger prospective studies involving patients from different settings and with longer follow-up periods are warranted to confirm our findings.

To conclude, our study has revealed distinctive attributes among young individuals with DD, identifying specific risk factors and demonstrating a unique disease trajectory characterized by a more aggressive course both at diagnosis and follow-up. These findings highlight the importance of implementing preventive policies among the younger population, especially focusing on lifestyle, diet, smoking, and alcohol habits, to mitigate the disease rate. Furthermore, they emphasize the importance of an accurate diagnosis to start prompt and appropriate therapeutic interventions, thereby averting surgical complications. In this regard, personalized medical and surgical management for diverticulitis along with preventive measures to minimize the recurrence should be considered key cornerstones for managing DD in young patients.

## Data availability statement

The raw data supporting the conclusions of this article will be made available by the authors, without undue reservation.

## Ethics statement

The studies involving humans were approved by IRCCS San Matteo Hospital Foundation, 2016, Protocol number 004820. The studies were conducted in accordance with the local legislation and institutional requirements. The participants provided their written informed consent to participate in this study.

## Author contributions

GS: Conceptualization, Data curation, Formal analysis, Investigation, Methodology, Writing – original draft, Writing – review & editing. MLe: Conceptualization, Data curation, Funding acquisition, Methodology, Project administration, Supervision, Writing – review & editing. GAb: Investigation, Writing – review & editing. GAl: Investigation, Writing – review & editing. FD: Investigation, Writing – review & editing. CF: Investigation, Writing – review & editing. AG: Investigation, Writing – review & editing. MLa: Investigation, Writing – review & editing. CP: Investigation, Writing – review & editing. DR: Investigation, Writing – review & editing. SS: Investigation, Writing – review & editing. NA: Investigation, Writing – review & editing. ADe: Data curation, Formal analysis, Software, Writing – review & editing. GC: Supervision, Writing – review & editing. MI: Supervision, Writing – review & editing. ADi: Conceptualization, Data curation, Funding acquisition, Methodology, Project administration, Supervision, Writing – review & editing.
